# Angle Insensitive Color Filters in Transmission Covering the Visible Region

**DOI:** 10.1038/srep19289

**Published:** 2016-01-14

**Authors:** Kening Mao, Weidong Shen, Chenying Yang, Xu Fang, Wenjia Yuan, Yueguang Zhang, Xu Liu

**Affiliations:** 1 State key laboratory of Modern Optical Instrumentation, Department of Optical Engineering, Zhejiang University, Hangzhou 310027, China

## Abstract

Angle insensitive color filter based on Metal-SiO_x_-Metal structure is proposed in this paper, which can keep the same perceived transmitted color when the incidence angle changes from 0° to 60°, especially for p-polarization light. Various silicon oxide films deposited by reaction magnetron sputtering with a tunable refractive index from 1.97 to 3.84 is introduced to meet the strict angle insensitive resonance conditions. The angle resolved spectral filtering for both p-polarization light and s-polarization light are quite well, which can be attributed to the different physical origins for the high angular tolerance for two polarizations. Finally, the effect of SiO_x_ absorption and Ag thickness on the peak transmittance are analyzed.

Color filters, involving from chemical filters with organic dyes to optical filters including thin film filters and nano-structured filters based on specific nanostructures, e.g. grating arrays and random surface have played an important role in enormous applications such as imaging sensors, liquid crystal display, decorations and solar cells^1^,^2^,^3^,^4^,^5^. Dye based chemical filters widely used in liquid crystal display, will have significant performance degradation when exposed to longstanding ultraviolet illumination and high temperature^4^. Though the multilayer interference filters have good spectral filtering properties with high reflection/transmission, they have to face the challenge that the color produced by (HL)s film stack is quite sensitive to the incidence angle^6^. Such angle dependent spectral characteristic makes it difficult to apply the thin film color filters for the applications under wide cone-angle incidence. Therefore, angle insensitive structural color with different nanostructure attracts great interests and has been extensively researched for these decades^7^,^8^. However, it is time-consuming and high-cost for the large area manufacturing of those nanostructured filters with the processes of e-beam lithography or focused ion beam, which prevents these structural colors from practical applications^9^.

Fabry-Perot (FP) type resonators with a dielectric layer sandwiched by two metal films have been widely studied for spectrum filtering^10^,^11^. The typical FP structure faces the same challenge of blue-shift as most thin film optical filters do. But the angular tolerance of metal-dielectric-metal (MDM) FP structure can be greatly improved if some particular conditions are satisfied^12^,^13^,^14^, especially for the matching condition of material characteristics. The rigorous conditions (n = κ, n and κ are respectively refractive index of the dielectric material and extinction coefficient of metal material) highly restrict the constitution of the MDM structure. Limited to the material condition of the metal and the dielectric, only isolated samples with specific colors were demonstrated^12^,^15^. To achieve the spectrum filtering over the whole visible light region, silicon oxide (SiO_x_) with a tunable refractive index is introduced in our study to meet the strict demands of the materials. Silicon oxide deposited by reactive magnetron sputtering was adjusted by controlling the pressure of oxygen during deposition to satisfy the resonance condition with the metal silver. Color filters based on this structure comprised of silver (Ag) and specific silicon oxide (SiO_x_) were fabricated which can present the same perceived transmitted color up to 60 degrees. The angle resolved spectral filtering for p-polarization light is as well as that for s-polarization, which can be attributed to different physical origins for two polarizations. Finally, the effects of SiO_x_ absorption and Ag thickness on the peak transmittance are analyzed.

## Results

**Figure 1:**
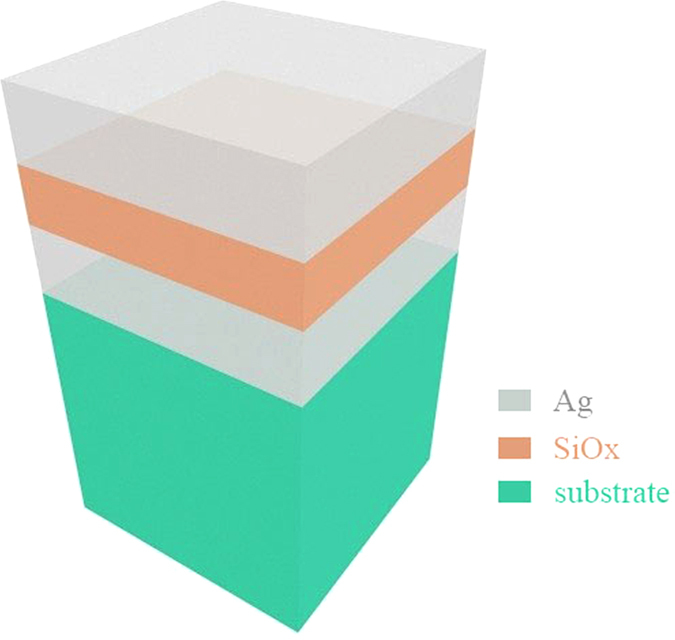
The MDM structure with SiO_x_ film sandwiched by Ag layers.

**Figure 2:**
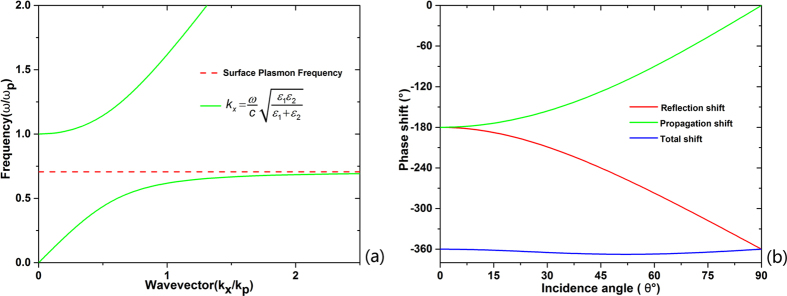
(**a**) Dispersion curve of a MDM structure comprising two Ag mirrors separated by SiO_x_ with the optical thickness of λ_sp_/4. (**b**) The round-trip phase shift in the MDM structure. The red line shows the reflection phase shift at the two interfaces and the green line shows the propagation phase shift in the surface normal in the dielectric. The total round-trip phase shift is represented by the blue line which is approximately 360° for all the incidence angles.

In our study, the basic structure consists of two semi-infinite metallic films spaced by a dielectric layer, shown in Fig. 1. Ag is chosen as the metal layer for its low optical absorption loss at visible frequencies. The dispersion curve of the MDM structure is shown in Fig. 2(a) while TM mode (p-polarization light) is considered. The lossless Drude model (ε_2_ = 1 − ω_p_^2^/ω^2^, where ω_p_ is the bulk plasma frequency of the metal) is adopted to describe the dielectric function of the metallic layers. Both metal and dielectric are presumed nonmagnetic (μ_1_ = μ_2_ = 1). It can be seen from Fig. 2(a) that the light propagating in the MDM structure has three modes: radiative mode, quasi-bound mode and propagating surface plasmon polariton (SPP) mode from top to bottom. Here we mainly focus on the quasi-bound mode. When the parallel part of the wave vector κ approaches infinity, ω(κ) (the dispersion frequency as a function of κ) tends towards ω_sp_, which is the surface plasmon frequency at the metal-spacer interface. At this frequency, the optical constant of metal is equal in magnitude and opposite in sign to that of the dielectric:


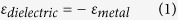


In this case, with the proper thickness of the dielectric layer


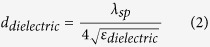


the frequency at κ = 0 will coincide with ω_sp_, (λ_sp_ = 2πc/ω_sp_ is the free-space surface plasmon wavelength) then the dispersion band of quasi-bound mode may become almost completely flat. Therefore, such a flat dispersion band indicates that the resonance occurs approximately at the same wavelength for all incidence angles.

The angular insensitive phenomenon of this structure for p-polarization can also be explained by Fabry-Perot resonant cavity theory. As shown in Fig. 2(b) the round-trip phase shift in the MDM structure includes two parts: the propagation phase shift accumulated when the light wave goes through the dielectric region and the reflection phase shift at the metal–dielectric interfaces. If Equation (1) and (2) are satisfied, the aggregate reflection phase shift at the metal-spacer interfaces almost exactly cancels the phase shift due to the propagation of the wave in the direction perpendicular to the interfaces in the spacer region and the total phase shift reaches approximately 2π for all incident angles^12^,^15^.

**Figure 3:**
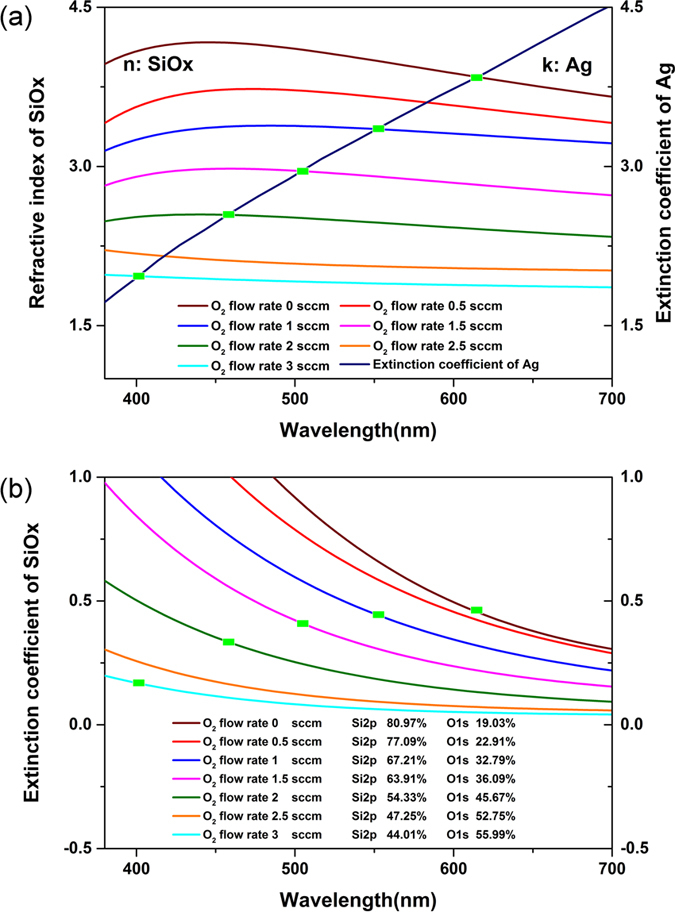
(**a**) The optical constant of the SiO_x_ film deposited by reactive sputtering and the Ag material. The real part of refractive index of SiO_x_ and the extinction coefficient of Ag is considered. The optical constant of Ag comes from Palik^18^. According to the resonance condition of material, the intersections of the SiO_x_ index and the Ag extinction coefficient indicates the resonance case. (**b**) The extinction coefficient of SiO_x_ with different content of Si. The green dots are corresponded with the resonance case shown in (**a**).

As known to us, Ag is a good conductive metal and the real part of its refractive index is quite small at visible region. So the relative permittivity of Ag is approximately described as ε_Ag_ = −κ_Ag_^2^, κ_Ag_ is the imaginary part of refractive index of Ag. For a typical dielectric material, the imaginary part of their refractive index is close to 0, leading to ε_dielectric_ = n^2^. Therefore, to achieve the angle insensitive properties for p-polarized light, the refractive index of the dielectric material should be equal to κ_Ag_. At visible region, Ag presents anomalous dispersion properties. As shown in Fig. 3(a), the extinction coefficient of Ag increases monotonously from 1.9 to 4.5 as the wavelength increases from 400 nm to 700 nm. According to n = κ_Ag_, dielectric layers with the matching refractive index are necessary to fabricate the angle insensitive MDM filters. Unfortunately, the refractive index of dielectric material commonly used in the visible region for optical coatings is limited to 2.5. Thus, only filters with resonant wavelength less than 480 nm were fabricated, e.g., ZnS(n = 2.5) at the resonant wavelength of 474 nm for transmission type and SiO_2_(n = 1.46) at the resonant wavelength of 352nm for reflection type^12^,^15^.

Silicon as a semiconductor material is widely used to manufacture the mid-infrared optical filters. It has a high refractive index (n > 4.5) in visible region which is a good candidate to construct the angle insensitive MDM filter with Ag. Moreover, SiO_x_ film can be achieved if oxygen gas is introduced during the deposition process of silicon. The refractive index of SiO_x_ determined by the composition of the material (the oxygen content in the SiO_x_ film) can be continuously tuned by controlling the deposition parameters. Theoretically, SiO_x_ can turn into pure amorphous silicon with no oxygen or silicon dioxide with sufficient oxygen as the critical case^16^,^17^. Therefore, the angle insensitive filters at various resonant wavelengths over the whole visible region can be obtained.

Reactive magnetron sputtering is used to deposit the SiO_x_ films in our study. The silicon with the purity 99.999% is the target. The substrate is a double polished fused silica with 15 × 15 mm and the substrate is kept at room temperature during the deposition. The distance between the target and substrate is 15cm. The chamber is pumped to a base vacuum pressure 5 × 10^−5^ Pa. Argon and Oxygen are introduced respectively as the sputtering and reactive gas. The technical parameters including the sputtering power, the deposition vacuum pressure and the oxygen flow rate will affect the optical properties. Among these parameters, oxygen flow rate has a largest impact on the optical constant of the SiO_x_ film. Thus, the SiO_x_ film with different refractive index was fabricated by tuning the oxygen/ argon flow rate ratio. The Ar flow rate is set at 90 sccm, the deposition pressure is 2 × 10^−2^ Pa and the sputtering power is kept at 400 w.

The optical constants of the SiO_x_ film shown in Fig. 3(a), were determined by the photometry method by fitting the measured reflectance and transmittance curves. The detailed fitting method and fitting qualityare provided in the supplementary information. The refractive index of SiO_x_ in the visible region is gradually reduced as the O2 flow rate is increased. The refractive index@550 nm reduces from 4.2 to1.7 when the O2 flow rate changes from 0 sccm to 3 sccm. The composition of the films characterized by XPS(X-ray photoelectron spectroscopy) is shown in Fig. 3(b). As O2 flow rate is increased, the content of oxygen in SiO_x_ film is raised up owing to the reaction between Si and O2 during the sputtering deposition. For comparison, the extinction coefficient of Ag is also plotted in Fig. 3(a). According to the resonance condition, κ_Ag_ = n_SiOx_ can be deduced from Equation (1) if the extinction coefficient of SiO_x_ film is ignored. It implies that the intersection between the Ag extinction coefficient curve and the SiO_x_ index curve indicate a perfect resonance at the selected wavelengths. Therefore, the green dots marked in Fig. 3 are the ideal resonant points. To construct an insensitive color filter at a specific central wavelength, a SiO_x_ film with a specific oxygen content and refractive index is required. By adjusting the oxygen flow rate during the deposition, the refractive index of SiO_x_ films can be varied from 1.46 to 4.2. So, the resonance condition will be satisfied with tunable SiO_x_ film covering the whole visible light region and various colors can be obtained with this method as desired. In our study, five color filters with different central wavelengths as indicated in Fig. 3(a) are experimentally manufactured. The corresponding refractive index, thickness and oxygen flow rate during the deposition of SiO_x_ layer at the given resonance wavelengths are listed in Table 1.

For the transmission filters, the absorption caused by the spacer layer must be considered. As is shown in Fig. 3(b), all the SiO_x_ films show a normal dispersion in the visible region. The extinction coefficient of different SiO_x_ fims decreases as the content of oxygen in the film increases, i.e., a lower refractive index of the SiO_x_ film leads to a lower absorption. For the selected central wavelengths of the angle insensitive filters shown as the green dots in Fig. 3, the extinction coefficient of the corresponding SiO_x_ film decreases as the central wavelength turns shorter. Thus, the filter with a shorter central wavelength will have a relative higher peak transmittance.

**Figure 4:**
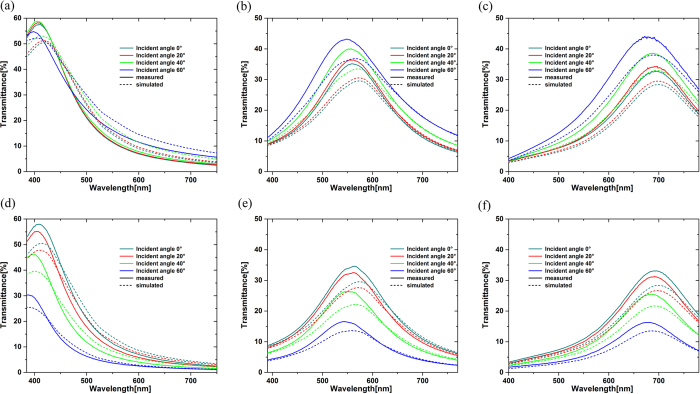
The simulated and measured transmittances. The different colored lines represents different incidence angle from 0° to 60°. The solid lines represents the measured results while the dot lines for the simulated results. (**a–c**) p-polarization transmittance for devices designed at the designed resonant wavelength of 399.1 nm, 506.9 nm and 614.9 nm respectively. (**d–f**) s-polarization transmittance for devices designed at the designed resonant wavelength of 399.1 nm, 506.9 nm and 614.9 nm respectively.

**Figure 5:**
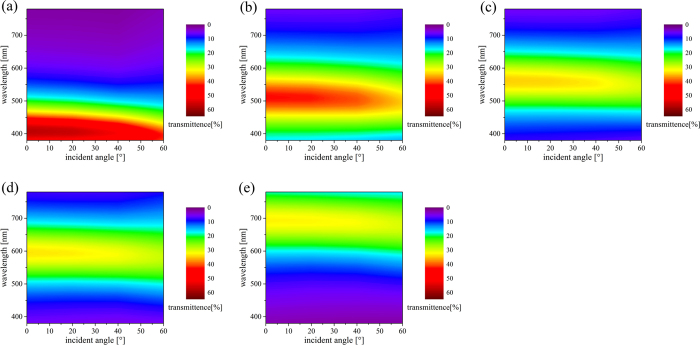
The measured angle resolved transmission at unpolarized incidence of the fabricated devices when the incident angle varies from 0° to 60°. The resonant wavelengths (399.1 nm, 456.2 nm, 506.9 nm, 551.4 nm and 614.9 nm) are in accordance with Table 1 and Fig. 6.

The angle resolved transmission spectra of these devices calculated by transfer matrix method as well as the measured results are shown in Fig. 4(a–c) for p-polarization^6^. The angle resolved transmittance of these devices were measured by the spectrophotometer (Shimadzu UV-3101PC) with a rotating sample stage. For p-polarization light, it is obvious that good angle insensitivity is remained for all these fabricated filters up to 60 degrees, matching well with the simulation results and confirming the theoretical analysis. Generally, the optical property of these color devices for s-polarization must be taken into account as well for its practical application. Figure 4(d–f) show the simulated and measured angle resolved transmission of these devices for s-polarization. It is not hard to find that the angle insensitive property for s-polarization is not bad, which could be ascribed to the high refractive index of SiO_x_^10^. When the incidence angle in air increases to 60°, the refraction angle in SiO_x_ layer is still small, which leads to a small resonance shift. The small difference of the resonant wavelengths between the simulated and the measured are caused by the errors of the sputtering conditions and the thickness of Ag layers. For the fabricated devices the peak transmittance for both two polarizations decreases as the resonant wavelength goes towards long wavelength, this is because the corresponding extinction coefficient of SiO_x_ is increased, which can be seen in Fig. 3(b). The peak transmittance reaches a stable level of 30% as the resonant wavelength increases. This is because when the extinction coefficient increases, the refractive index increases as well. According to Equation (2), the physical thickness of SiO_x_ films with longer resonance wavelength are thinner than that of films with shorter resonance wavelength, which is shown in Table 1. Therefore, the total absorption caused by SiO_x_ films can be restrained and be kept at an acceptable level. While the incidence angle increases, the peak transmittance of p-polarization light will be increased and the s-polarization light decreased, thus leads to a constancy for the average transmittance, shown in Fig. 5. For unpolarized incidence, the peak transmittance of all these filters is higher than 32%, which provides a guarantee for the practical applications of this transmission filters. And the half-band width (about 100 nm) is relatively wide for the thin Ag layers which could not provide enough reflectance but the peak transmittance and the half-band width must be balanced. So the MDM angle insensitive filters with tunable SiO_x_ is available for non-polarized case and can be potentially applied for diverse colorful applications. In order to demonstrate the color change quantitatively, the spectral data of average transmittance are transformed into CIEDE2000 Color-Difference Chart and are shown in Table 2. It can be seen that the color change is not large for all the five filters.

It is worth mentioning that the thickness of the Ag layer carries a huge impact on the optical properties of the proposed filters, which influences not only the peak transmittance of the filters, but also the angle insensitive characteristics and the resonant wavelengths. When the Ag layers are thick enough (at least 30 nm), perfect angle insensitive characteristics (less than 5 nm resonant wavelength shift at the incidence angle of 60°) will be obtained, especially for p-polarization, however the peak transmittance will be decreased to less than 10% at red spectral band. When the thickness of the Ag layers decreases, the peak transmittance will be increased greatly but the filters will have a decline in angle insensitive properties and the center resonant wavelengths will have a red shift compared with the designed position. Therefore, thickness of 16 nm is selected for the Ag layers balance the peak transmittance and the angle insensitive characteristic.

**Figure 6:**
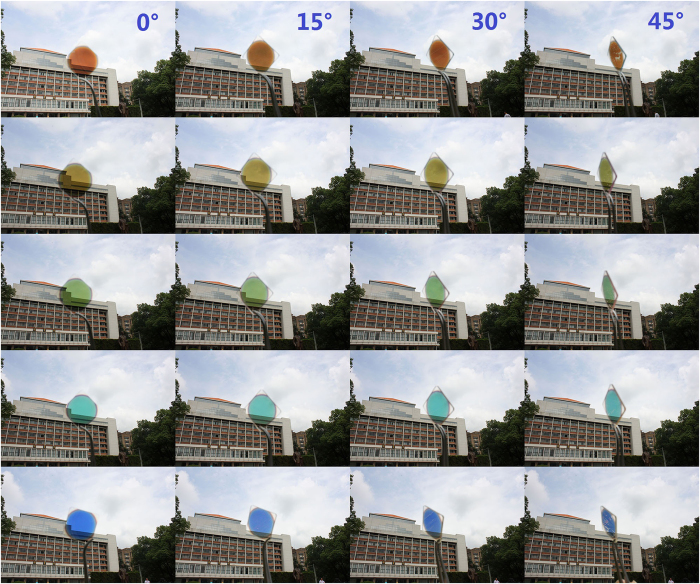
The photo of the fabricated devices taken at different incident angles 0°, 15°, 30°, 45°.

Figure 6 is a photo image of the fabricated devices taken with outdoor ambient light under sunshine at four different angles up to 45°. The back ground is clearly seen through the devices owing to the relatively high peak transmittance and when the devices are rotated from 0° to about 45°, both the transmittance and the color change very little. Hence, the proposed devices are fabricated only through vacuum deposition and well performed can have enormous potential in applications where angle insensitive characteristic are needed.

## Discussion

In conclusion, a compact film structure based on MDM resonator is proposed to achieve the efficient angle insensitive color filtering for transmission spectrum across the whole visible light wavelengths. Silicon oxide deposited by reaction magnetron sputtering with a tunable refractive index is introduced to meet the strict rigorous conditions demands of the materials. Color filters fabricated in our study can present the same perceived color up to 60 degrees with the peak transmittance of over 32%, which agrees well with the simulation results. The angle resolved spectral filtering for p-polarization light is as well as that for s-polarization, which can be attributed to the different physical origins for the high angular tolerance for two polarizations. Moreover, the transmittance can be optimized by controlling the thickness of Ag layers. Furthermore, gold can be adopted in such structure for the infrared region. The method has enormous potential in applications of display, remote sensing, decoration, and anti-counterfeiting.

## Methods

### Simulation

Simulation of the transmittance of the fabricated filters was performed by the transfer matrix method. In our simulation, the optical constants of Ag was from the data of Palik^18^ and the refractive indexes, extinction coefficient and thickness of SiO_x_ were experimentally determined by spectrometry method considering both the reflectance and the transmittance curves. The details are provided in the supplementary information.

### Device fabrication

The proposed color filters were manufactured on a double polished fused silica with 15 × 15 mm. The film stacks were all deposited by magnetron sputtering with the base vacuum pressure better than 5 × 10^−5^ Pa. During the deposition, the substrates were kept at room temperature. The distance between the target and substrate is 15cm. For SiO_x_, the Ar flow rate is set at 90sccm, the deposition pressure is 2 × 10^−2^ Pa and the sputtering power is kept at 400w. For Ag the Ar flow rate is set at 40sccm, the deposition pressure is 1.5 × 10^−1^ Pa and the sputtering power is kept at 70 w.

### Optical characterization

The reflectance/transmittance measurement was performed by the spectrophotometer (Shimadzu UV-3101PC).

## Additional Information

**How to cite this article**: Mao, K. *et al.* Angle Insensitive Color Filters in Transmission Covering the Visible Region. *Sci. Rep.* **6**, 19289; doi: 10.1038/srep19289 (2016).

## Supplementary Material

Supplementary Information

## Figures and Tables

**Table 1:** Parameters of the angle insensitive filters in our study in accordance with Fig. 3.

Wavelength[nm]	O2 flow rate[sccm]	SiO_x_ refractive index	Ag extinction coefficient	SiO_x_ thickness[nm]	Ag thickness[nm]
399.1	3	1.967	1.967	50.7	18
456.2	2	2.545	2.545	44.8	16
506.9	1.5	2.954	2.954	42.9	16
551.4	1	3.352	3.352	41.1	16
614.9	0	3.843	3.843	40.1	16

**Table 2:** CIEDE2000 Color-Difference in transmission at different incident angles.

CIE2000	399.1 nm	456.2 nm	506.9 nm	551.4 nm	614.9 nm
0°	0	0	0	0	0
20°	1.97829	1.97851	1.28366	1.45319	0.812718
40°	4.64326	7.09077	4.1612	5.29557	2.24258
60°	10.0045	15.8292	8.55313	11.9436	6.63285
