# Supplementary material for: Angle Insensitive Color Filters in Transmission Covering the Visible Region
Source: Sci Rep. 2016 Jan 14;6:19289. doi: 10.1038/srep19289 (PMC4725860; doi:10.1038/srep19289)
Supplement: Supplementary Information [file srep19289-s1.doc]

<Supplementary Information>

**Angle Insensitive Color Filters in Transmission Covering the Visible Region**

Kening Mao, Weidong Shen*, Chenying Yang, Xu Fang, Wenjia Yuan, Yueguang Zhang, and Xu Liu

* E-mail: adongszju@hotmail.com

State key laboratory of Modern Optical Instrumentation,

Department of Optical Engineering, Zhejiang University, Hangzhou 310027, China

**The fitting method and fitting quality**

“The fitting is done under the help of optical coating software OptiChar. The dispersion law of SiO_x_ is selected as the arbitrary dispersion model. During the fitting procedure, the two conditions should be satisfied: n and k should be continuous at different wavelength; and the physical thickness should be the same for all wavelengths. Least square method is used to find out the best solutions. When the optical constants and physical thickness have been determined, refractive index dispersion of SiO_x_ can be described by$n(\lambda)=a+b/\lambda^{2}+c/\lambda^{4}$. The dispersions for different SiOx films are shown in Table S1.

| O_2_ flow rate [sccm] | a | b | c |
| --- | --- | --- | --- |
| 0 | 2.719067 | 0.57649684 | -0.05725717 |
| 0.5 | 2.652375 | 0.4813566 | -0.05373186 |
| 1 | 2.77382 | 0.28614329 | -0.03351742 |
| 1.5 | 2.210225 | 0.32343027 | -0.03399747 |
| 2 | 1.972582 | 0.22195539 | -0.02139994 |
| 2.5 | 1.973085 | 0.01818175 | 0.00234002 |
| 3 | 1.793962 | 0.03562601 | -0.0012843 |

Table S1 The parameters of the refractive index dispersion for different SiO_x_ films


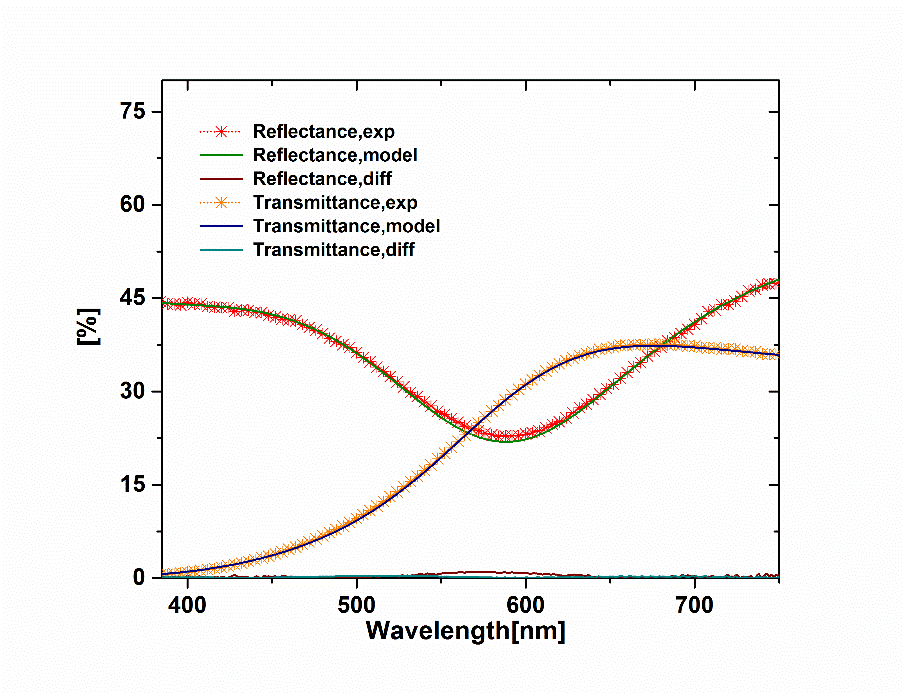


Fig. S1 (a) The difference of reflectance and transmittance between the experimental and simulated data for SiO_x_ film when O_2_=0sccm


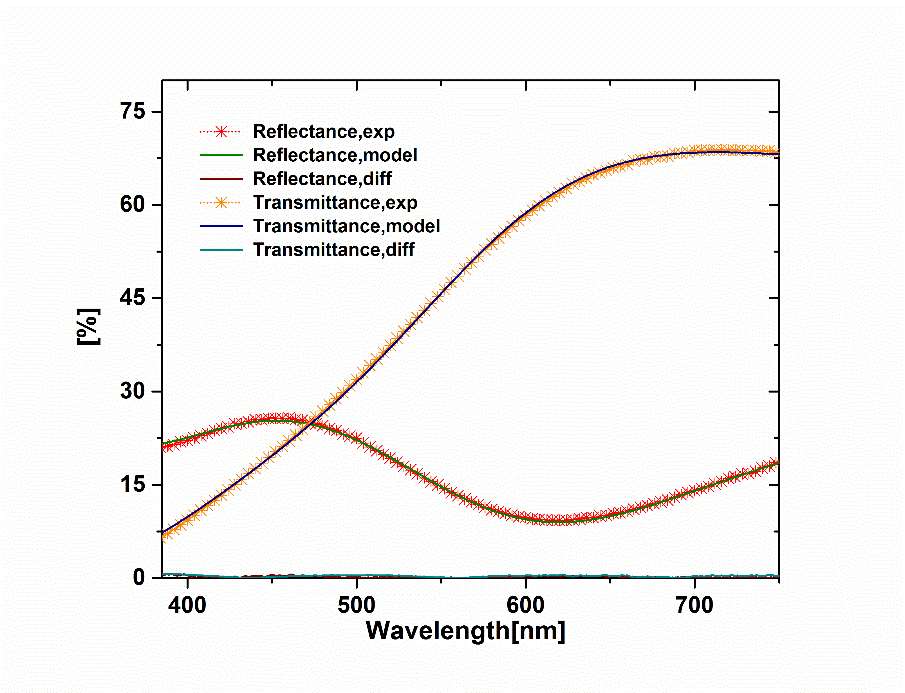


Fig. S1 (b)The difference of reflectance and transmittance between the experimental and simulated data for SiO_x_ film when O_2_=2sccm

Two fitting examples are shown in Figure S1 for two SiOx films with 0 and 2sccm oxygen. The solid lines are the simulated data and the star lines are the experimental data. The curves in Figure S1 show that the simulated curves agree well with those experimental curves. The differences between the simulated and experimental data are very small.

**Optical constants of the Ag thin layer**

The optical constant of thin Ag layer is determined by fitting the measured reflectance and transmittance curves at the angle of incidence 0^0^ and 45^0^. As shown in Figure S2, the middle lines are data at 6°and the others are respectively data for P and S polarizations at 45°. The quality of the fitting between the experimental and simulated curves is also quite well. The optical constants of the Ag layer at the thickness of 16nm and 23nm are shown in Fig. S3. It can be seen that refractive index and extinction coefficient for 16nm and 23nm layers agree well with the data from Palik.

**
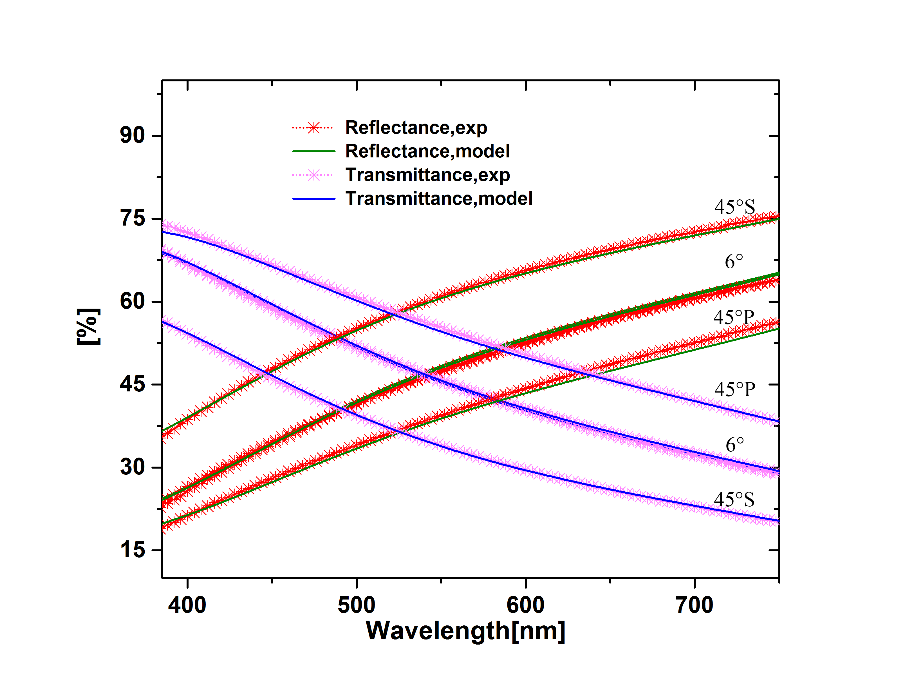
**

Fig. S2 The measured and simulated transmittance and reflectance of Ag thin layers


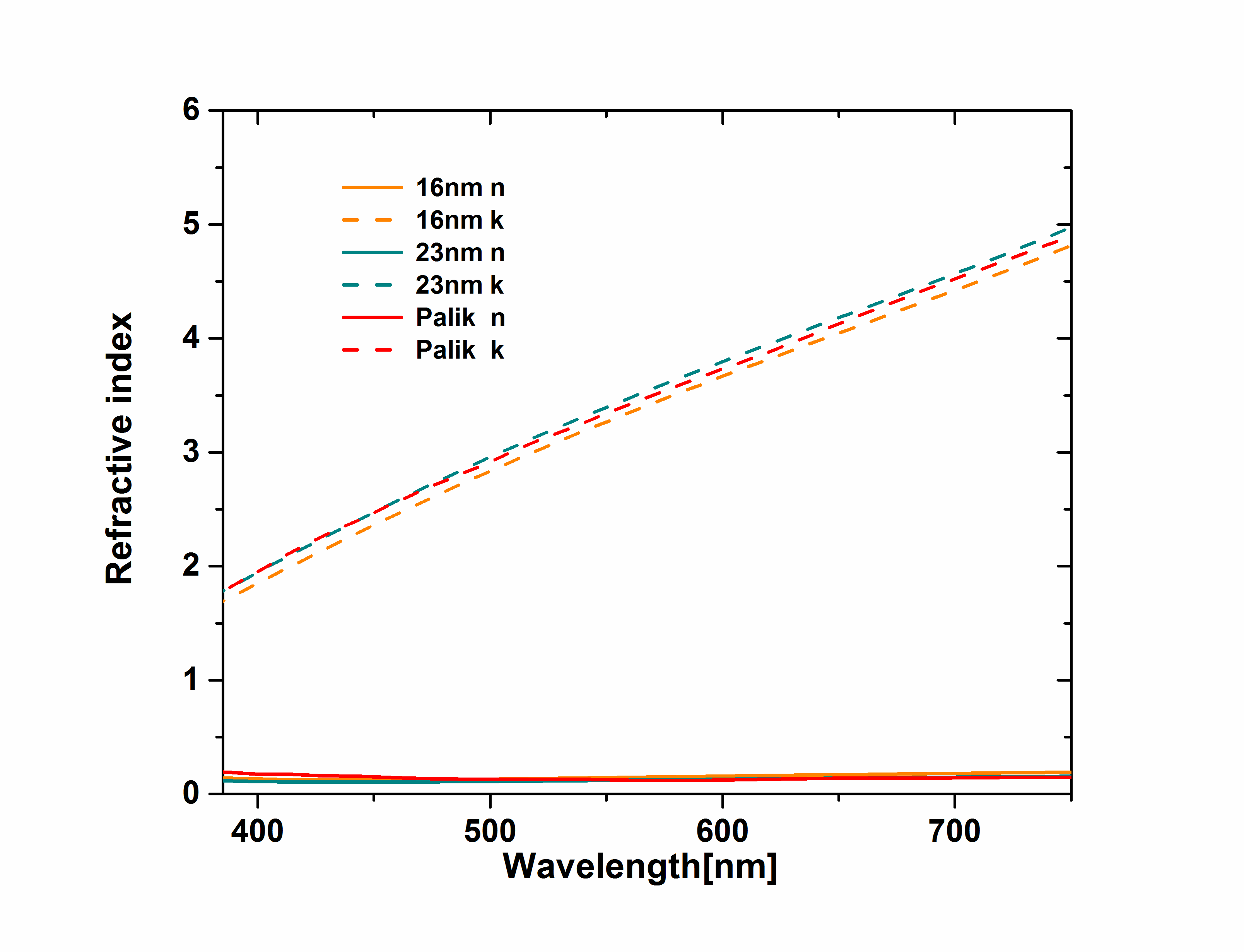


Fig. S3 Optical constants of the Ag layers and the comparison with those from Palik
